# Efficacy and safety of systematic corticosteroids among severe COVID-19 patients: a systematic review and meta-analysis of randomized controlled trials

**DOI:** 10.1038/s41392-021-00521-7

**Published:** 2021-02-21

**Authors:** Shaolei Ma, Changsheng Xu, Shijiang Liu, Xiaodi Sun, Renqi Li, Mingjie Mao, Shanwu Feng, Xian Wang

**Affiliations:** 1 Department of Emergency and Critical Care Medicine, Zhongda Hospital Affiliated to Southeast University, Nanjing, China; 2 Department of Anesthesiology, The First Affiliated Hospital of Nanjing Medical University, Nanjing, China; 3 Department of Pain Medicine, The First Affiliated Hospital of Nanjing Medical University, Nanjing, China; 4 Department of Anesthesiology, Jinling Hospital, School of Medicine, Nanjing University, Nanjing, China; 5 Department of Anesthesiology, Women’s Hospital of Nanjing Medical University, Nanjing Maternity and Child Health Care Hospital, Nanjing, China

**Keywords:** Immunotherapy, Infectious diseases

## Abstract

The benefits and harms of corticosteroids for patients with severe coronavirus disease 2019 (COVID-19) remain unclear. We systematically searched PubMed, Embase, and Cochrane Central Register of Controlled Trials from December 31, 2019 to October 1, 2020 to identify randomized controlled trials (RCTs) that evaluated corticosteroids in severe COVID-19 patients. The primary outcome was all-cause mortality at the longest follow-up. Secondary outcomes included a composite disease progression (progression to intubation, ventilation, extracorporeal membrane oxygenation, ICU transfer, or death among those not ventilated at enrollment) and incidence of serious adverse events. A random-effects model was applied to calculate risk ratio (RR) with 95% confidence intervals (CIs). We used the Grading of Recommendations Assessment, Development, and Evaluation approach to evaluate the certainty of the evidence. Seven RCTs involving 6250 patients were included, of which the Randomized Evaluation of COVID-19 Therapy (RECOVERY) trial comprised nearly 78% of all included subjects. Results showed that corticosteroids were associated with a decreased all-cause mortality (27.3 vs. 31.1%; RR: 0.85; 95% CI: 0.73–0.99; *P* = 0.04; low-certainty evidence). Trial sequential analysis suggested that more trials were still required to confirm the results. However, such survival benefit was absent if RECOVERY trial was excluded (RR: 0.83; 95% CI: 0.65–1.06; *P* = 0.13). Furthermore, corticosteroids decreased the occurrence of composite disease progression (30.6 vs. 33.3%; RR: 0.77; 95% CI: 0.64–0.92; *P* = 0.005), but not increased the incidence of serious adverse events (3.5 vs. 3.4%; RR: 1.16; 95% CI: 0.39–3.43; *P* = 0.79).

## Introduction

Resulted from severe acute respiratory syndrome coronavirus 2 (SARS-CoV-2), coronavirus disease 2019 (COVID-19) is a global life-threatening pandemic. As estimated, ~20% of the COVID-19 patients will advance to acute respiratory distress syndrome (ARDS).^1^ Cytokine and chemokine storms are considered to participate in the development of such respiratory, or even multi-organ failure (MOF), leading to the application of immunosuppressive drug such as corticosteroids in clinical practice. However, the efficacy and safety of corticosteroids in COVID-19 patients is currently not conclusive based on available evidence.

Recently, the Randomized Evaluation of COVID-19 Therapy (RECOVERY) collaborative suggested a prominent survival benefit of corticosteroid treatment with a low dosage of dexamethasone daily for up to 10 days in subjects receiving oxygen therapy or mechanical ventilation, especially favoring those receiving mechanical ventilation.^2^ Upon the release of this promising report, several ongoing randomized controlled trials (RCTs) stopped enrollment and published their results in advance, such as the REMAP-CAP trial,^3^ CAPE COVID trial,^4^ and CoDEX randomized clinical trial.^5^ However, these trials did not consistently report definite benefits for corticosteroid treatment. In the REMAP-CAP trial, compared with no hydrocortisone, a 7-day fixed-dose or shock-dependent dosage of hydrocortisone lead to 93 and 80% likelihood of superiority for reduction in organ support-free days, not meeting expected criteria for statistical superiority.^3^ While, in the CAPE COVID trial, compared to placebo, low-dose hydrocortisone did not reduce mortality or the requirement for respiratory support.^4^

Furthermore, after the release of the RECOVERY report, the World Health Organization (WHO) Rapid Evidence Appraisal for COVID-19 Therapies (REACT) Working group performed a prospective meta-analysis with the last follow-up date being July 7, 2020, encompassing 7 RCTs and 1703 critically ill COVID-19 patients, and suggested that compared with usual care or placebo, systematic corticosteroid treatment was related to a lower 28-day all-cause mortality (odds ratio (OR): 0.66; 95% confidence interval (CI): 0.53–0.82).^6^ However, this conclusion was thereafter questioned to be highly dependent on the RECOVERY trial, which comprised 59% (*n* = 1007) patients.^7^ If the RECOVERY trial was excluded, the magnitude of association between corticosteroids and mortality decreased prominently (OR: 0.78; 95% CI: 0.56–1.07). Meanwhile, the RECOVERY trial has uncertainties, including a high mortality that cannot be generalized to other settings, absence of results for long-term prognosis, and corticosteroids risk.

Considering the aforementioned uncertainties, this systematic review and meta-analysis of corticosteroids includes more recent rigorous RCTs to address the efficacy and safety of corticosteroids among severe COVID-19 patients.

## Methods

This systematic review and meta-analysis was performed in consistent with Preferred Reporting Items for Systematic Reviews and Meta-Analyses (PRISMA) statement.^8^ The protocol is prospectively registered on PROSPERO (CRD42020210851).

### Inclusion and exclusion criteria

Eligible trials need to meet the PICO criteria (participants, intervention, comparator, and outcome). Participants were adults, with laboratory-confirmed (positive SARS-CoV-2 polymerase chain reaction from nasopharyngeal swabs, sputum samples, or bronchoalveolar lavage samples) or clinically suspected (suggestive computed tomography scan finding in the absence of any other cause of pneumonia) COVID-19. The intervention included any kind of corticosteroids in combination with standard, usual care, compared with standard, usual care, or placebo alone, without corticosteroids. Notably, we enrolled only severe COVID-19 patients. The definition of “severe” is specified according to lung injury severity, varying among trials, overall, minimally requiring respiratory support at randomization in this meta-analysis, including those receiving oxygen therapy, invasive or noninvasive mechanical ventilation, or extracorporeal membrane oxygenation (ECMO).

The primary outcome was all-cause mortality at the longest follow-up, defined by the individual trial. The secondary outcomes included a composite disease progression and the incidence of serious adverse events during treatment. The definition of composite disease progression and serious adverse events may slightly differ among trials. For this meta-analysis, we did not try to standardize but used the prespecified definition. Trials performed among “non-severe” participants or with primary outcome not available were excluded.

### Literature search

Eligible RCTs were identified with a comprehensive systematic search of databases including PubMed, Embase, and Cochrane Central Register of Controlled Trials, from December 31, 2019 to October 1, 2020. Search terms were first applied with “corticosteroid” OR “hormones, adrenal cortex” OR “corticoids.” The search results were then combined with “COVID-19” OR “2019 novel coronavirus disease” OR “COVID19” OR “COVID-19 virus disease” OR “2019 novel coronavirus infection” OR “2019-nCoV infection” OR “coronavirus disease 2019” OR “coronavirus disease-19” OR “2019-nCoV disease” OR “COVID-19 virus infection.” The results were limited to humans with no language restrictions. Detailed search strategy is shown in Table S1. Finally, reference lists of the retrieved papers and reviews relating to corticosteroids treatment for COVID-19 were screened as well to minimize omissions.

### Data extraction

Two investigators (S.M. and C.X.) independently extracted data from the eligible trials with disagreement resolved by discussion or consultation with another reviewer (S.L.). The following data were collected: first author, year of publication, abbreviation of each trial, region of trial, trial type, inclusion criteria, timing, dosage, and duration of corticosteroids, control intervention, the observed primary outcome in each trial, as well as the time-point of longest follow-up.

### Risk of bias

Two independent investigators (S.L. and X.S.) performed risk assessment with the Cochrane Collaboration risk of bias,^9^ which comprises with seven distinct domains: random sequence generation, allocation concealment, blinding of participants and personnel, blinding of outcome assessment, incomplete outcome data, selective reporting, and other bias. Each domain was categorized as “low,” “unclear,” or “high” risk. The highest rated bias for each domain was deemed as the risk of bias for the trial.

### Statistical analysis

Meta-analysis was conducted with RevMan 5.3 software (Nordic Cochrane Center). The Mantel–Haenszel method was applied to calculate risk ratio (RR) and 95% CI with a random-effects model. Forest plots showed the pooled results. A *P* value < 0.05 was considered to have statistical significance. Heterogeneity across trials was assessed with the *I*^*2*^ statistic. In this study, an *I*^*2*^ of 25–50%, 50–75%, and >75% was suggested to have low, moderate, and high heterogeneity, respectively. *I*^*2*^ > 50% suggests significant heterogeneity.^10^

For the primary outcome, subgroup analyses were planned according to corticosteroids dosage and treatment duration, both of which were clinical considerations during corticosteroids treatment. A low or high dosage was defined based on the cutoff values: dexamethasone 15 mg/day, hydrocortisone 400 mg/day, or equivalent methylprednisolone 80 mg/day.^11^ Besides, treatment duration was classified as <7 or ≥7 days. The subgroup analysis was performed only for all-cause mortality due to the small number of trials for other outcomes.

### Trial sequential analysis (TSA) for all-cause mortality

Cumulative meta-analysis may produce type I error (false positive) because of random errors or systematic bias. TSA controls the risk of type I and type II errors via estimating the trial sequential monitoring boundaries and the required sample size.^12^ If the resulted Z curve crosses the trial sequential monitoring boundary or futility boundary, the anticipated intervention effect is suggested to be confirmative.^13^ However, if the Z curve does not cross the abovementioned boundaries or not reach the required information size, current evidence is not sufficient to draw a definite conclusion.

We determined the required information size with a two-sided 5% risk for type I error (α) and 20% risk for type II error (β) with a relative risk (RR) reduction 20% in all-cause mortality. The control event proportion was obtained from the no corticosteroids group. TSA version 0.9 beta software was applied (http://www.ctu.dk/tsa).

### Evidence quality assessment

Two reviewers (R.L. and M.M.) assessed the evidence certainty based on the Grading of Recommendations Assessment, Development, and Evaluation (GRADE) framework.^14^ Evidence quality was downgraded based on five domains including risk of bias, inconsistency, indirectness, imprecision, and publication bias. Collectively, the evidence certainty was rated as very low, low, moderate, and high.

## Results

### Trials selection

**Fig. 1:**
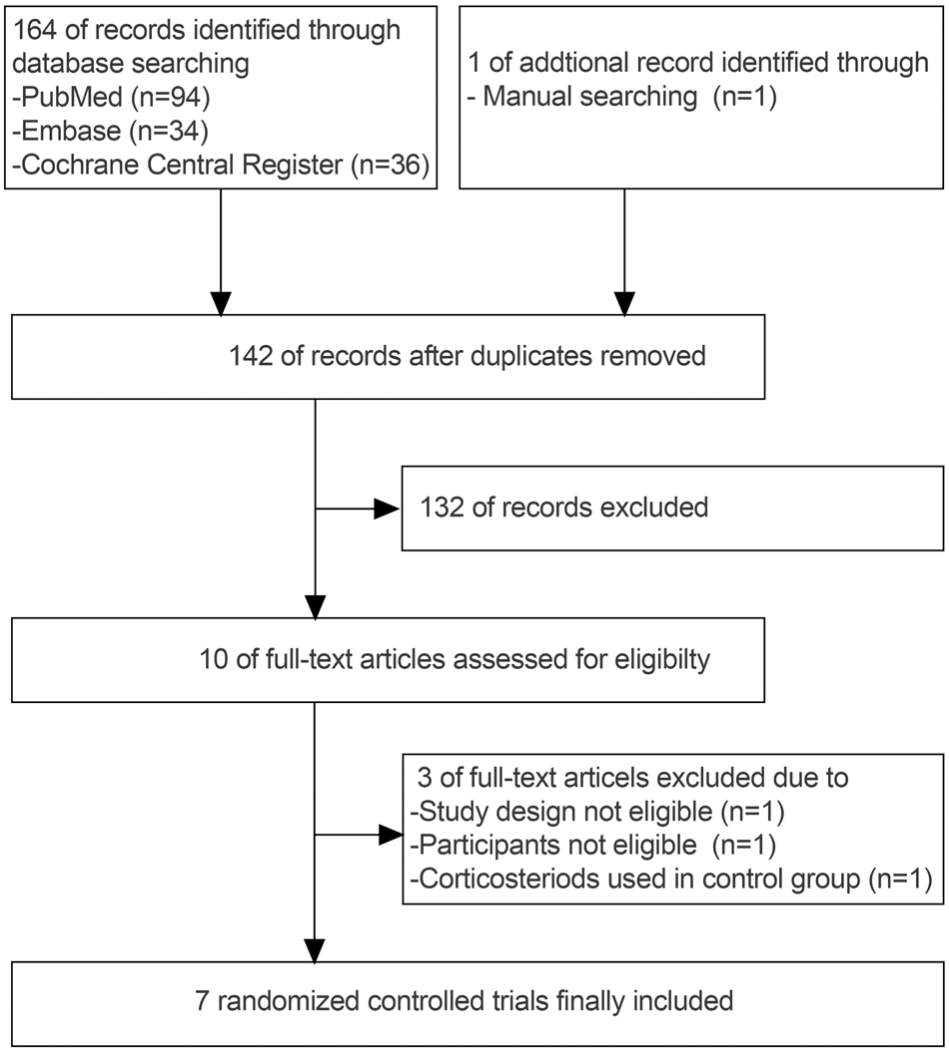
PRISMA flowchart for the identification, screening, eligibility, and inclusion of trials

We initially identified 164 records from databases and another 1 by manual searching. After removing 23 duplicates and another 132 records by screening the title and abstract, there were 10 full-text articles left. Three articles were further excluded for not meeting the inclusion criteria. Then, seven eligible trials were finally included in this meta-analysis.^2–5,15–17^ The selection flow diagram is shown in Fig. 1.

### Trials characteristics

The main characteristics of the included trials are shown in Table 1. All trials were available in 2020. The sample size ranged from 62 to 4890 (total 6250; 2385 receiving corticosteroids and 3865 receiving standard or usual care without corticosteroids). Of the included trials, one was single center^17^ and six were multicenter trials.^2–5,15,16^ The corticosteroids used included hydrocortisone,^3,4^ dexamethasone,^2,5^ and methylprednisolone.^15–17^ Five trials applied a low dosage^2–4,15,17^ and two trials^5,16^ used a high dosage of corticosteroids. Besides, corticosteroids was used with a duration < 7 days in three trials^15–17^ and ≥7 days^2–5^ in another four trials, respectively.

**Table 1:** Characteristics of included trials

Trial	Region of trial	Trial type	Inclusion criteria	Timing of corticosteroids	Dosage and duration of corticosteroids (*n*)	Control intervention (*n*)	Primary outcome in each trial	Longest follow-up
Angus et al.^3^ REMAP-CAP	Australia, Canada, France, Ireland, the Netherlands, New Zealand, the UK, the USA	Multicenter, open-label, RCT	Aged at least 18 years	Given at study day 1 [1–4]	A fixed 7-day course of intravenous hydrocortisone (50 or 100 mg every 6 h) (*n* = 137)^a^ OR A shock-dependent course (50 mg every 6 h up to 28 d for shock patients) (*n* = 141)	Usual care, no hydrocortisone (*n* = 101)	Respiratory and cardiovascular organ support-free days to 21 d	21 d
			Confirmed or suspected COVID-19					
			Admitted to ICU receiving respiratory or cardiovascular support					
Corral et al.^15^ GLUCOCOVID	Spain	Multicenter, partial randomized, preference, open-label controlled trial	Aged at least 18 years	Not specified	Methylprednisolone 80 mg/d for 3 d, then 40 mg/d for 3 d (*n* = 49)^b^	Standard of care, no corticosteroids (*n* = 29)	A composite of death, ICU admission, or requirement of noninvasive ventilation	Until composite endpoint happened
			Confirmed COVID-19					
			Severe pneumonia, not intubated or ventilated					
Dequin et al.^4^ CAPE COVID	France	Multicenter, RCT	Aged at least 18 years	Within 24 h of the onset of the first severity criterion or within 48 h for patients referred from another hospital	Hydrocortisone 200 mg/d for 7 d, then 100 mg/d for 4 d and 50 mg/d for 3 d; if symptoms improved by day 4, then followed with hydrocortisone 100 mg/d for 2 d and 50mg/d for 2 d (*n* = 76)	Standard care (*n* = 73)	Death or persistent respiratory support on 21 d	21 d
			Confirmed or suspected COVID-19					
			Admitted to ICU with acute respiratory failure					
Edalatifard et al.^16^	Iran	Multicenter, single-blind, RCT	Aged at least 18 years	Not specified	Methylprednisolone 250 mg/d for 3 d (*n* = 34)	Standard care (*n* = 28)^b^	Time to clinical improvement and hospital discharge or death	Until clinical improvement and hospital discharge or death
			Confirmed COVID-19					
			Receiving oxygen therapy but not intubation or ventilation					
Horby et al.^2^ RECOVERY	UK	Multicenter, open-label, RCT		Not specified	Oral or intravenous dexamethasone 6 mg/d for up to 10 d (or until hospital discharge if sooner) (*n* = 1603)	Usual care (*n* = 3287)	All-cause mortality within 28 d after randomization	28 d
			Confirmed or suspected COVID-19					
			Received respiratory support^c^					
Jeronimo et al.^17^ Metcovid	Brazil	Single center, RCT	Aged at least 18 years	Not specified	Methylprednisolone 1 mg/kg/d for 5 d (*n* = 194)	Placebo (*n* = 199)	Mortality at 28 d	28 d
			Confirmed or suspected COVID-19					
			In use of oxygen therapy or under invasive mechanical ventilation					
Tomazini et al.^5^ CoDEX trial	Brazil	Multicenter, open-label, RCT	Aged at least 18 years	Not specified	Dexamethasone 20 mg/d for 5 d, then 10 mg/d for 5 d or until ICU discharge (*n* = 151)	Standard care (*n* = 148)	Ventilator-free days at 28 d	28 d
			Confirmed or suspected COVID-19					
			Receiving mechanical ventilation for ARDS					

^a^Only two subjects were assigned 100 mg every 6 h for 7 days

^b^Based on per-protocol analysis

^c^This trial also included patients not requiring oxygen therapy

### Risk of bias

Risk of bias of the seven included trials is summarized in Figs. S1 and S2 and Table S2. As indicated, one trial has low risk of bias,^17^ while the other six trials^2–5,15,16^ are considered to have a high risk due to reasons such as open-label or single-blind study design, protocol deviation, or an early stop to enrollment.

### Primary outcome: all-cause mortality

**Fig. 2:**
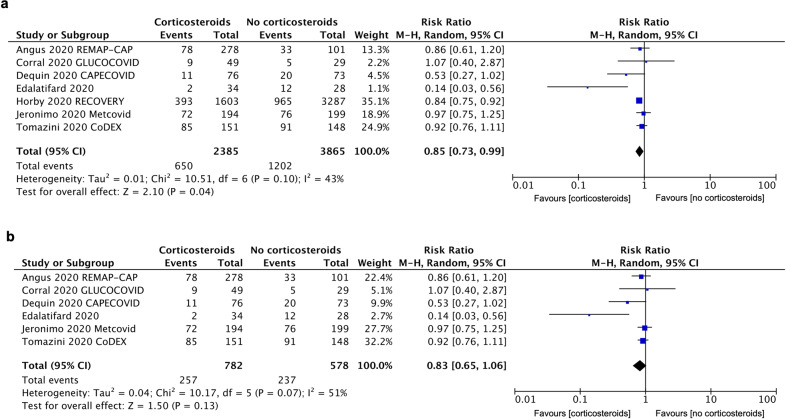
Forest plot comparing corticosteroids treatment vs. no corticosteroids on all-cause mortality in severe COVID-19 patients. **a** Forest plot of all-cause mortality including all the seven trials. **b** Forest plot of all-cause mortality without RECOVERY trial. M–H Mantel–Haenszel, CI confidence interval, df degrees of freedom

All included trials reported data on the all-cause mortality. There were 650 deaths among 2385 patients receiving corticosteroids (27.3%) and 1202 deaths among 3865 patients not receiving corticosteroids (31.1%). The pooled RR was 0.85 (95% CI: 0.73–0.99, *P* = 0.04), with a low heterogeneity (*I*^*2*^ = 43%) (Fig. 2a). As shown in Figs. S3 and S4, the benefit was observed in a low dosage of corticosteroids (RR: 0.85; 95% CI: 0.77–0.93; *P* = 0.0003; *I*^*2*^ = 0%) and treatment duration ≥ 7 days (RR: 0.85; 95% CI: 0.78–0.92; *P* = 0.0001; *I*^*2*^ = 0%). However, if RECOVERY trial excluded, such survival benefit was absent in the remaining six trials (RR: 0.83; 95% CI: 0.65–1.06; *P* = 0.13; *I*^*2*^ = 51%) (Fig. 2b).

**Fig. 3:**
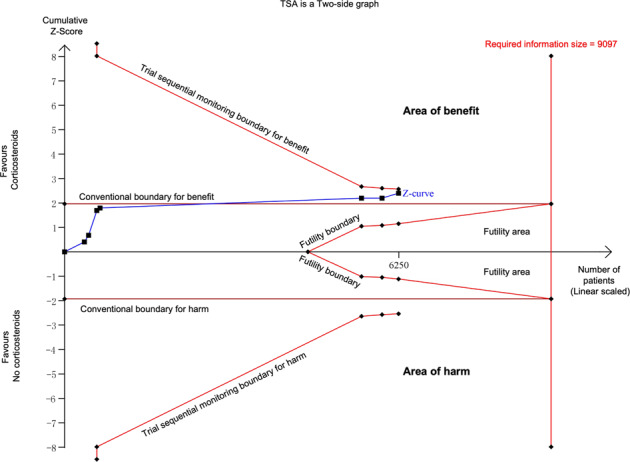
TSA for all-cause mortality comparing corticosteroids vs. no corticosteroids. TSA was performed with control arm event (no corticosteroids) proportion of 31.1%, relative risk reduction of 20%, α of 5% (two sided), and β of 20%. The required information size was obtained as 9097. Z curve crossed the conventional boundary but not crossed the trial sequential monitoring boundary; meanwhile, it not reached the required information size

As shown by TSA results, the cumulative Z curve crossed the conventional boundary but not crossed the trial sequential monitoring boundary; meanwhile, it not reached the required information size of 9097 (Fig. 3). Thus, additional trials might be required although current evidence suggesting a benefit for all-cause mortality.

### Secondary outcomes

Four trials including 4161 patients reported disease progression^2–4,15^; however, the definition of “progression” differed among trials, from development to invasive mechanical ventilation or death,^2^ to endotracheal intubation,^4^ progression to intubation, ECMO, or death,^3^ or to ICU transfer, intubation, or death.^15^ Finally, there were 448 progressions among 1464 patients receiving corticosteroids (30.6%) and 899 progressions among 2697 patients not receiving corticosteroids (33.3%). The pooled RR for a composite disease progression was found to be 0.77 (95% CI: 0.64–0.92; *P* = 0.005; *I*^*2*^ = 47%) (Fig. S5).

Besides, four trials reported serious adverse events,^3–5,16^ of which 19 events were reported for 539 patients receiving corticosteroids and 12 for 350 patients not receiving corticosteroids. Reported serious adverse events varied among trials; however, the risk of serious adverse events was not increased in those receiving corticosteroids (RR: 1.16; 95% CI: 0.39–3.43; *P* = 0.79; *I*^*2*^ = 31%) (Fig. S6).

### Evidence quality of the outcomes in this meta-analysis

Based on GRADE methodology, evidence quality was low for all-cause mortality, a composite disease progression, as well as the incidence of adverse events (Table 2).

**Table 2:** Summary of findings for outcomes comparing corticosteroids vs. no corticosteroids

No. of studies	Quality assessment					Relative effect (95% CI)	Absolute effect	Quality
	Risk of bias	Inconsistency	Indirectness	Imprecision	Publication bias			
All-cause mortality								⊕⊕⊝⊝ LOW
7	Serious^a^	None	None	None	Suspected^b^	RR 0.85 (0.73–0.99)	47 fewer per 1000 (from 3 fewer to 84 fewer)	
A composite disease progression								⊕⊕⊝⊝ LOW
4	Serious^a^	None	None	None	Suspected^b^	RR 0.85 (0.77–0.93)	50 fewer per 1000 (from 23 fewer to 77 fewer)	
Serious adverse events								⊕⊕⊝⊝ LOW
4	Serious^a^	None	None	None	Suspected^b^	RR 1.13 (0.54–2.38)	4 more per 1000 (from 16 fewer to 47 more)	

^a^Some included studies have high risk of bias according to risk of bias results

^b^Due to small number of included trials, publication bias cannot be excluded

## Discussion

In the present meta-analysis including 7 RCTs and 6250 severe COVID-19 patients, corticosteroids treatment was related to a reduction of all-cause mortality and disease progression, but not with an increase in serious adverse events. However, such survival benefit was absent if RECOVERY trial excluded. TSA confirmed the finding in all-cause mortality but also suggested that additional RCTs still required. Together with great heterogeneity among trials and low evidence certainty, a definite conclusion remains a challenge.

COVID-19-related respiratory failure or MOF might result from excessive host immune response that injured pulmonary alveoli, resulting in a cytokine and chemokine storm along with systemic damage. To dampen such inflammatory dysfunction, the use of corticosteroids has attracted great attention. Corticosteroids have been used in ARDS resulting from SARS-CoV-1 or Middle East respiratory syndrome-CoV, both having manifestation of diffuse alveolar damage and pulmonary inflammatory injury.^18,19^ Nevertheless, there is no consensus in the literature that corticosteroids provide definite benefits for COVID-19 patients, considering the possibility of a delay in the virus clearance and increased secondary infections or adverse events, such as hyperglycemia, psychosis, or avascular necrosis.

Several meta-analytical studies have evaluated the efficacy of corticosteroids for COVID-19 patients with mixed results. Meta-analysis by Pei et al.^20^ included 5 retrospective studies in 943 patients and concluded that corticosteroids use might increase risk of death (OR: 2.43; 95% CI: 1.44–4.1; *P* = 0.0001). Similarly, in another meta-analysis based on cohort studies and case series, Cheng et al.^21^ also concluded that corticosteroids were not effective to decrease mortality, shorten duration of symptoms, or virus clearance time. Of note, both of these two meta-analyses included non-RCTs but observational studies, which had diverse confounding or bias, such as residual confounding, survivor bias, treatment selection bias, collectively decreasing the quality of the conclusion. Conversely, in a recent prospective meta-analysis from the REACT Working group, compared with usual care or placebo, systemic corticosteroids reduced the 28-day all-cause mortality for critically ill cases.^6^ Nonetheless, as thereafter queried by Tang et al.^7^, such a conclusion is highly dependent on the RECOVERY results. In addition, in a network meta-analysis comparing diverse drug interventions for COVID-19 up to July 20, direct pairwise meta-analysis for corticosteroids vs. standard care, which based on two RCTs (RECOVERY and GLUCOCOVID), indicated a probable decrease in mortality for corticosteroids (RR: 0.88; 95% CI: 0.80–0.97; risk difference 37 fewer per 1000 patients; moderate certainty), as well as a reduced progression to mechanical ventilation (RR: 0.74; 95% CI: 0.59–0.93; risk difference 30 fewer per 1000 patients, moderate certainty).^22^ However, if network meta-analysis applied, the pooled results would be less credible (0.84; 95% CI: 0.52–1.36 for mortality; and 0.71; 95% CI: 0.29–1.73 for mechanical ventilation).

Our meta-analysis suggests that corticosteroids reduced all-cause mortality compared with no corticosteroids in severe COVID-19 patients. Similar to Tang et al.’s^7^ finding, such survival benefit was absent if RECOVERY excluded (RR: 0.83; 95% CI: 0.65–1.06; *P* = 0.13). Furthermore, TSA for all-cause mortality showed current sample size did not reach the required information size with more additional trials required. Besides, it is noteworthy that the benefits of corticosteroids in severe viral respiratory infections highly depend on the selection of the right dosage and treatment duration, at the right timing, and in a specific subgroup of patients.

Subgroup analyses of different dosages and treatment duration were performed in this meta-analysis. Survival benefit was observed for a low dosage of corticosteroids (RR: 0.85, 95% CI: 0.77–0.93) and treatment duration not shorter than 7 days (RR: 0.85; 95% CI: 0.78–0.92). However, due to limited trials and great clinical heterogeneity, more data are required to elucidate the underlying clinical significance. The timing of corticosteroid therapy is another concern. Virus shedding in SARS-CoV-2 appears to peak on or before symptom onset and declines thereafter.^23^ Thus, theoretically, all enrolled severe patients in this meta-analysis were at the stage where the disease was dominated by immunopathological elements rather than active virus replication. During this period, corticosteroids could have a beneficial effect by reducing inflammatory storm, lung vascular permeability, alveolar edema fluid, and maintaining epithelial barrier integrity.^24^ The third contributing factor is the severity of COVID-19 disease. More robust evidence was obtained from the RECOVERY trial that dexamethasone provided survival benefit greatest in patients receiving invasive mechanical ventilation, followed by those receiving oxygen therapy without mechanical ventilation, but not in those who not requiring respiratory support at randomization.^2^ Consistently, in a recent meta-analysis encompassing COVID-19 patients with different disease severity, Pasin et al.^25^ suggested that corticosteroids might be considered in severe patients; however, discouraged for those mild subjects not receiving oxygen therapy.

The strengths of this meta-analysis contain a comprehensive search strategy to include high quality RCTs but not observational studies, the enrollment of all patients that require respiratory support to enhance generalizability, as well as rigorous application of the GRADE approach to evaluate evidence certainty. And for all-cause mortality, TSA was used to confirm the finding and calculate the required information size.

However, we also acknowledged that there are several limitations in this meta-analysis. First, significant clinical heterogeneity prominently weakened the results of this meta-analysis. Corticosteroids type, dosage, administration timing, treatment duration, as well as participants’ inclusion criteria and primary observational outcomes differed among trials, inevitably contributing to certain heterogeneity and requiring prudent interpretation of the pooled results. Second, current evidence did not provide a longer follow-up of corticosteroids for mortality or serious adverse events. Third, due to limited RCTs and heterogeneity among trials, it is currently not possible to perform meta-analysis for other outcomes such as organ support-free days, length of ICU or hospital stay, or duration of virus shedding. Fourth, our meta-analysis gave few clues as to which specific population would benefit greatest from corticosteroids treatment as subgroup analyses according to patients’ characteristics were not performed.

COVID-19 is a rapidly evolving global pandemic, with reliable treatment alternatives still unavailable. It is urgent to summarize the latest evidence to perform pooled analysis to guide clinical practice. After the RECOVERY report is released, the WHO updated guidelines recommend the use of corticosteroids in patients hospitalized with COVID-19.^26^ However, our meta-analysis suggests that it is still prudent to draw a definite conclusion with regard to the efficacy of corticosteroids for reducing all-cause mortality among severe COVID-19 patients. More robust supporting data are required.

## Conclusion

In this meta-analysis of 7 RCTs and 6250 severe COVID-19 patients, pooled results suggested that corticosteroids treatment was associated with a reduction in all-cause mortality and disease progression, but not an increase in serious adverse events comparing to no corticosteroids. However, the resulted survival benefit depended on the RECOVERY trial. And suggested by TSA, additional RCTs were required to confirm the efficacy of corticosteroids to reduce all-cause mortality. Together with great heterogeneity among trials and low evidence certainty, it remains prudent to draw a definite conclusion with regard to efficacy of corticosteroids among severe COVID-19 patients.

## Supplementary information

Supplementary material
