# Supplementary material for: Efficacy and safety of systematic corticosteroids among severe COVID-19 patients: a systematic review and meta-analysis of randomized controlled trials
Source: Signal Transduct Target Ther. 2021 Feb 21;6:83. doi: 10.1038/s41392-021-00521-7 (PMC7897363; doi:10.1038/s41392-021-00521-7)
Supplement: Supplementary file 1 — Supplementary material [file 41392_2021_521_MOESM1_ESM.docx]

Supplementary Material for

Efficacy and safety of systematic corticosteroids among severe COVID-19 patients: A systematic review and meta-analysis of randomized controlled trials

Shaolei Ma, Changsheng Xu, Shijiang Liu, Xiaodi Sun, Renqi Li, Mingjie Mao, Shanwu Feng, Xian Wang

Correspondence to: [iamfsw@163.com](mailto:iamfsw@163.com) OR [wangxian2002@126.com](mailto:wangxian2002@126.com)

**This PDF file includes:**

Figures. S1 to S6

Tables S1 to S2

**Figure. S1 Risk of bias summary: review authors’ judgement about each risk of bias item for included trials.**

Figure. S2 Risk of bias graph: review authors’ judgement about each risk of bias item presented as percentages across all included trials.


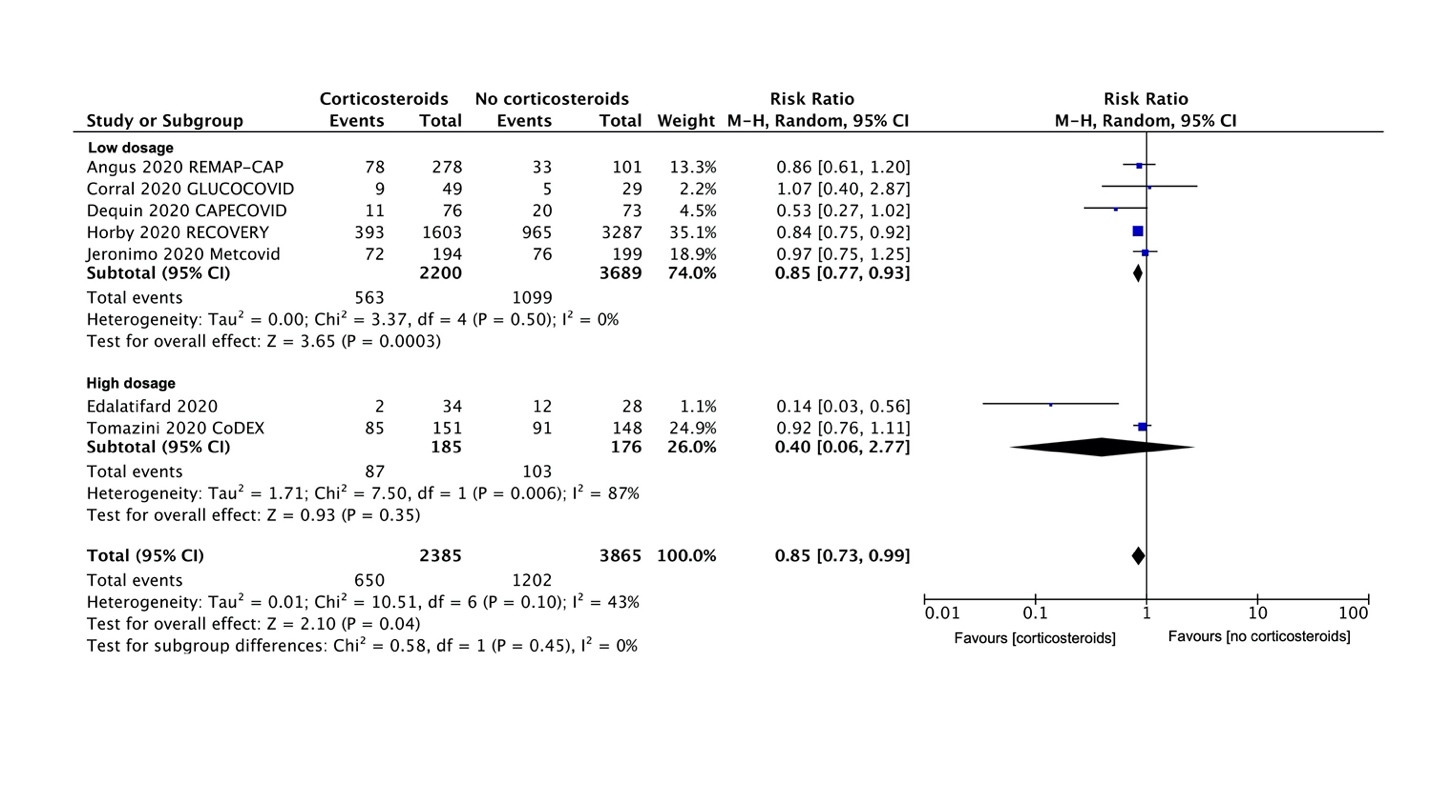


Figure. S3 Subgroup analysis for all-cause mortality based on corticosteroids dosage.


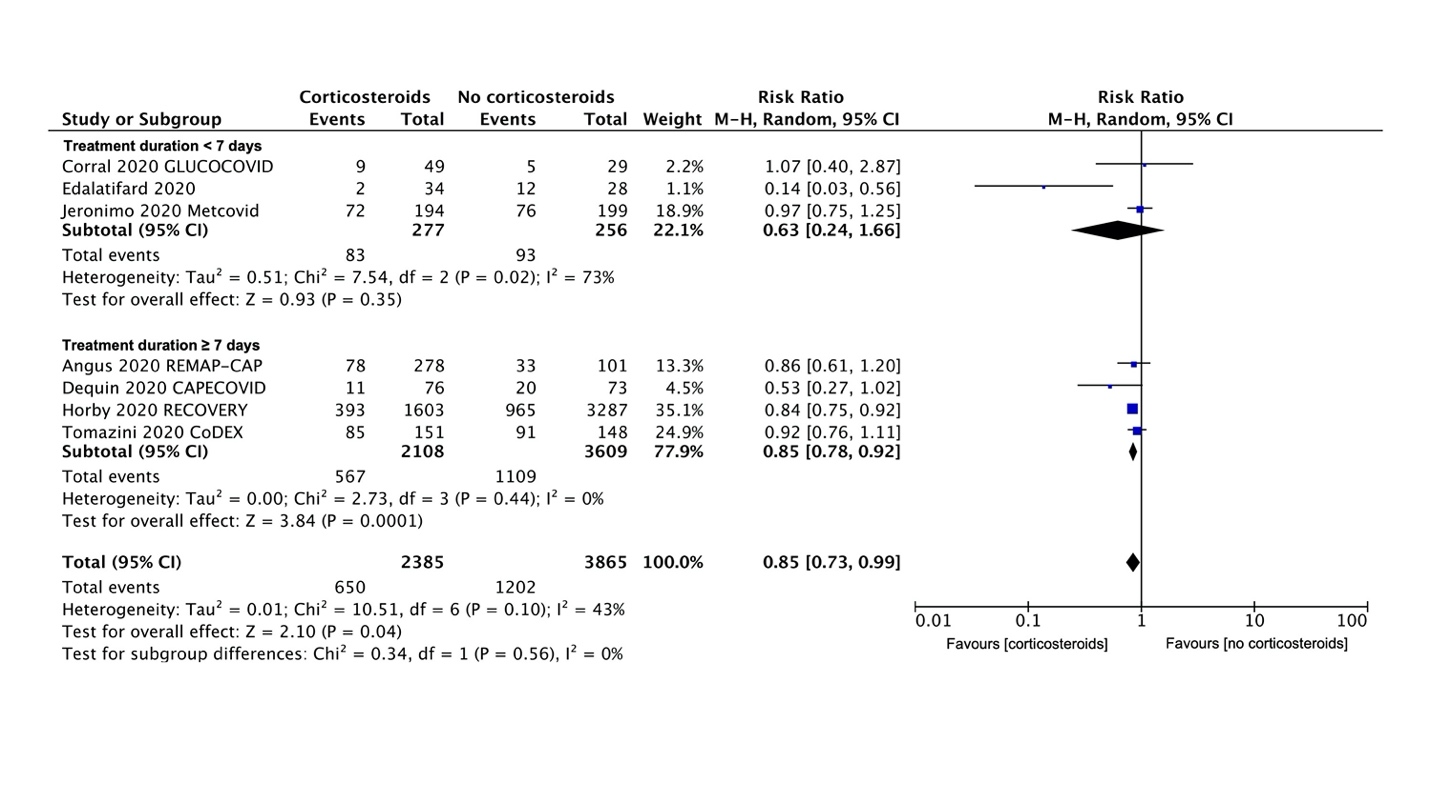


Figure. S4 Subgroup analysis for all-cause mortality based on corticosteroids treatment duration.


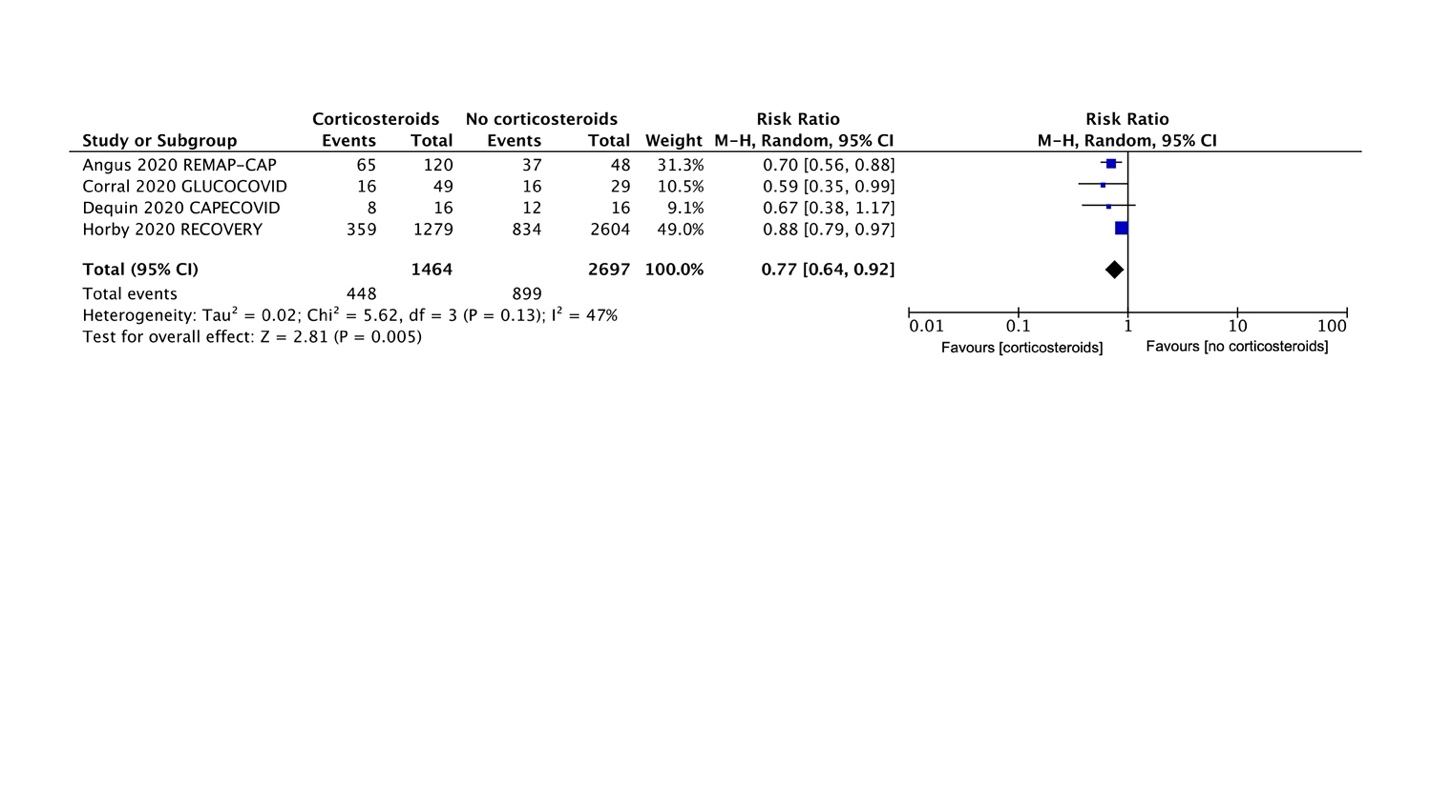


Figure. S5 Meta-analysis for a composite disease progression among patients receiving respiratory support but not ventilation at randomization.


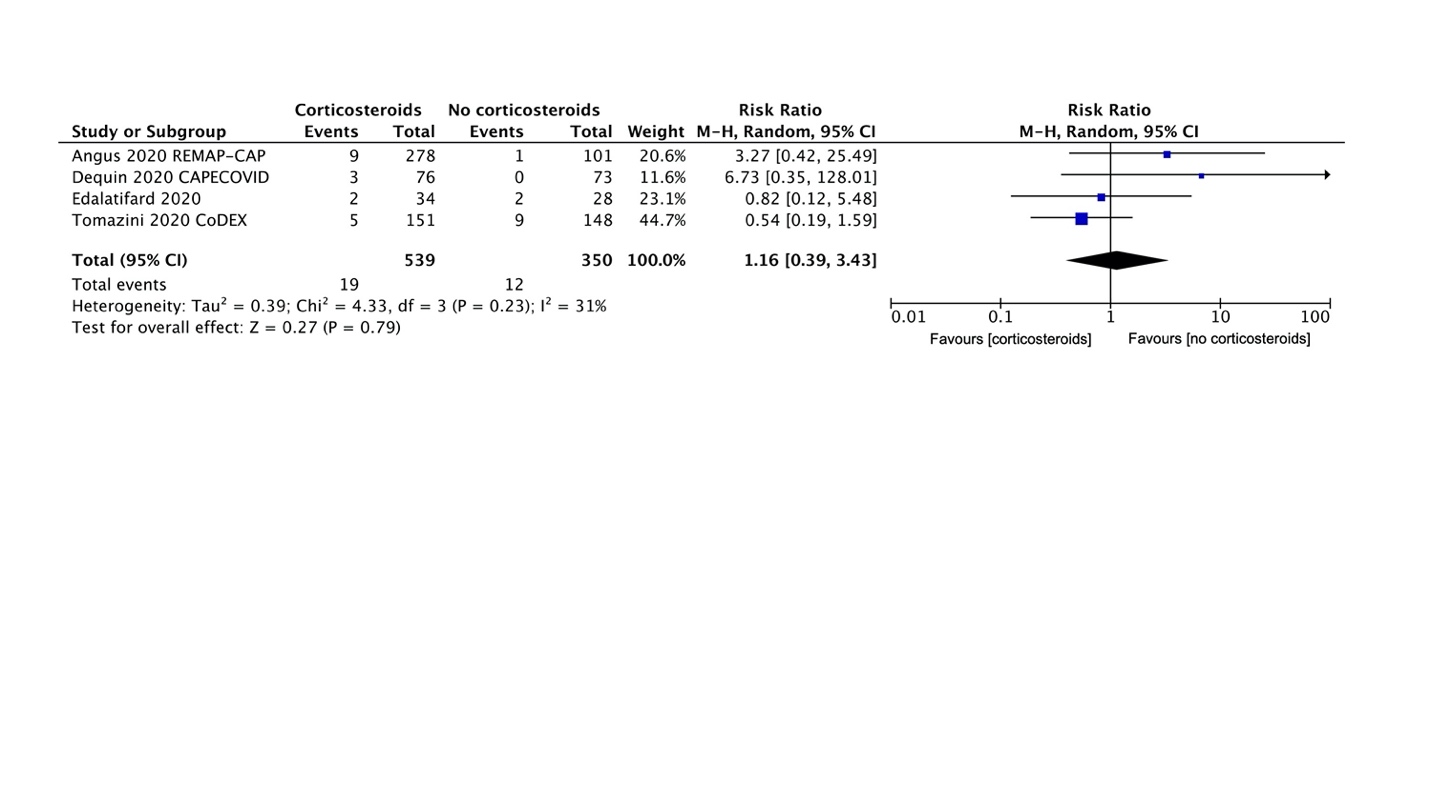


Figure. S6 Meta-analysis for serious adverse events.

Table S1

| **PUBMED** | | |
| --- | --- | --- |
| 1 | **(corticosteroid [MeSH Terms]) OR (((Hormones, Adrenal Cortex) OR (Corticosteroids)) OR (Corticoids))**  "adrenal cortex hormones"[MeSH Terms] OR ("adrenal cortex hormones"[MeSH Terms] OR ("adrenal"[All Fields] AND "cortex"[All Fields] AND "hormones"[All Fields]) OR "adrenal cortex hormones"[All Fields] OR ("hormones"[All Fields] AND "adrenal"[All Fields] AND "cortex"[All Fields]) OR "hormones adrenal cortex"[All Fields] OR ("adrenal cortex hormones"[MeSH Terms] OR ("adrenal"[All Fields] AND "cortex"[All Fields] AND "hormones"[All Fields]) OR "adrenal cortex hormones"[All Fields] OR "corticosteroid"[All Fields] OR "corticosteroids"[All Fields] OR "corticosteroidal"[All Fields] OR "corticosteroide"[All Fields] OR "corticosteroides"[All Fields]) OR ("adrenal cortex hormones"[MeSH Terms] OR ("adrenal"[All Fields] AND "cortex"[All Fields] AND "hormones"[All Fields]) OR "adrenal cortex hormones"[All Fields] OR "corticoid"[All Fields] OR "corticoids"[All Fields] OR "corticoides"[All Fields])) | 352560 |
| 2 | **(((((((((((COVID-19[MeSH Terms]) OR (2019 novel coronavirus disease)) OR (COVID19)) OR (COVID-19 pandemic)) OR (SARS-CoV-2 infection)) OR (COVID-19 virus disease)) OR (2019 novel coronavirus infection)) OR (2019-nCoV infection)) OR (coronavirus disease 2019)) OR (coronavirus disease-19)) OR (2019-nCoV disease)) OR (COVID-19 virus infection)**  "covid 19"[Supplementary Concept] OR "covid 19"[All Fields] OR "2019 novel coronavirus disease"[All Fields] OR "covid 19"[Supplementary Concept] OR "covid 19"[All Fields] OR "covid19"[All Fields] OR "covid 19"[Supplementary Concept] OR "covid 19"[All Fields] OR "covid 19 pandemic"[All Fields] OR "covid 19"[Supplementary Concept] OR "covid 19"[All Fields] OR "sars cov 2 infection"[All Fields] OR "covid 19"[Supplementary Concept] OR "covid 19"[All Fields] OR "covid 19 virus disease"[All Fields] OR "covid 19"[Supplementary Concept] OR "covid 19"[All Fields] OR "2019 novel coronavirus infection"[All Fields] OR "covid 19"[Supplementary Concept] OR "covid 19"[All Fields] OR "2019 ncov infection"[All Fields] OR "covid 19"[Supplementary Concept] OR "covid 19"[All Fields] OR "coronavirus disease 2019"[All Fields] OR "covid 19"[Supplementary Concept] OR "covid 19"[All Fields] OR "coronavirus disease 19"[All Fields] OR "covid 19"[Supplementary Concept] OR "covid 19"[All Fields] OR "2019 ncov disease"[All Fields] OR "covid 19"[Supplementary Concept] OR "covid 19"[All Fields] OR "covid 19 virus infection"[All Fields] | 58214 |
| 3 | **(((((((randomized controlled trial[Publication Type]) OR (controlled clinical trial[Publication Type])) OR (randomized[Title/Abstract])) OR (randomly[Title/Abstract])) OR (trial[Title/Abstract])) OR (placebo[Title/Abstract])) OR (groups[Title/Abstract])) OR (drug therapy[Title/Abstract])**  "randomized controlled trial"[Publication Type] OR "controlled clinical trial"[Publication Type] OR "randomized"[Title/Abstract] OR "randomly"[Title/Abstract] OR "trial"[Title/Abstract] OR "placebo"[Title/Abstract] OR "groups"[Title/Abstract] OR "drug therapy"[Title/Abstract] | 3149576 |
| 4 | 1# AND 2# AND 3# | 94 |
| **Embase** | | |
| 1 | 'corticosteroid'/exp | 1002142 |
| 2 | '11 hydroxycorticosteroid':ab,ti | 118 |
| 3 | '17 hydroxycorticosteroid':ab,ti | 352 |
| 4 | 'benzodrocortisone':ab,ti | 0 |
| 5 | 'corticosteroid':ab,ti | 70740 |
| 6 | 'corticosteroid derivative':ab,ti | 13 |
| 7 | 'glucocorticoid':ab,ti | 63581 |
| 8 | 'hydroxycorticosteroid':ab,ti | 488 |
| 9 | 'mineralocorticoid':ab,ti | 12077 |
| 10 | #1 OR #2 OR #3 OR #4 OR #5 OR #6 OR #7 OR #8 OR #9 | 1028923 |
| 11 | 'coronavirus disease 2019'/exp | 49174 |
| 12 | '2019 novel coronavirus disease':ab,ti | 184 |
| 13 | 'covid19':ab,ti | 45641 |
| 14 | 'covid-19 pandemic':ab,ti | 13052 |
| 15 | 'sars-cov-2 infection':ab,ti | 3331 |
| 16 | 'covid-19 virus disease':ab,ti | 0 |
| 17 | '2019 novel coronavirus infection':ab,ti | 50 |
| 18 | '2019-ncov infection':ab,ti | 129 |
| 19 | 'coronavirus disease 2019':ab,ti | 9283 |
| 20 | 'coronavirus disease-19':ab,ti | 532 |
| 21 | '2019-ncov disease':ab,ti | 9 |
| 22 | 'covid-19 virus infection':ab,ti | 13 |
| 23 | #11 OR #12 OR #13 OR #14 OR #15 OR #16 OR #17 OR #18 OR #19 OR #20 OR #21 OR #22 | 55673 |
| 24 | #10 AND #23 | 2133 |
| 25 | #24 AND 'randomized controlled trial'/de | 34 |
| **Cochrane Central Register of Controlled Trials** | | |
| 1 | MeSH descriptor: [Adrenal Cortex Hormones] explode all trees | 14361 |
| 2 | Hormones, Adrenal Cortex | 2498 |
| 3 | Corticosteroids | 13844 |
| 4 | Corticoids | 213 |
| 5 | #1 OR #2 OR #3 OR #4 | 25957 |
| 6 | (2019 novel coronavirus disease):ti,ab,kw | 88 |
| 7 | COVID19 | 117 |
| 8 | COVID-19 pandemic | 377 |
| 9 | SARS-CoV-2 infection | 72 |
| 10 | COVID-19 virus disease | 299 |
| 11 | 2019 novel coronavirus infection | 81 |
| 12 | coronavirus disease 2019 | 445 |
| 13 | coronavirus disease-19 | 23 |
| 14 | COVID-19 virus infection | 272 |
| 15 | #6 OR #7 OR #8 OR #9 OR #10 OR #11 OR #12 OR #13 OR #14 | 883 |
| 16 | #5 AND #15 | 36 |

Search strategy.

Table S2

| **Study** | **Bias** | **Support for judgement** |
| --- | --- | --- |
| Angus, 2020,  REMAP-CAP | Blinding of participants and personnel | Open-label design |
|  | Other bias | Early stop, protocol deviation (15% in the control group received corticosteroids for a short period) |
| Corral, 2020, GLUCOCOVID | Randomized sequence generation | Partially randomized |
|  | Blinding of participants and personnel | Open-label design |
| Dequin, 2020, CAPECOVID | Other bias | Early stop |
| Edalatifard, 2020 | Blinding of participants and personnel | Single blind design |
| Horby, 2020, RECOVERY | Blinding of participants and personnel | Open-label design |
| Tomazini, 2020, CoDEX | Blinding of participants and personnel | Open-label design |
|  | Other bias | Early stop, protocol deviation (35% in the control group received corticosteroids) |

Support for judgement for included trials rated as high risk of bias.
